# Pudgy mouse rib deformities emanate from abnormal paravertebral longitudinal cartilage/bone accumulations

**DOI:** 10.1242/bio.060139

**Published:** 2024-01-22

**Authors:** Frederic Shapiro, Jamie Wang, Evelyn Flynn, Joy Y. Wu

**Affiliations:** ^1^ Department of Medicine/Endocrinology, Stanford University School of Medicine, Palo Alto CA 94305, USA; ^2^ Department of Bioengineering, Northeastern University, Boston MA 02115, USA; ^3^ Orthopaedic Research Laboratory, Boston Children's Hospital, Boston MA 02115, USA

**Keywords:** Pudgy mouse, Abnormal rib formation, Supramolecular structural changes, Paravertebral tissue accumulation, Segmentation clock disorder

## Abstract

The pudgy (*pu/pu*) mouse*,* caused by a recessive mutation in the Notch family Delta like-3 gene (*Dll3*), has severe rib, vertebral body and intervertebral disc abnormalities. Using whole-mount preparations and serial histologic sections we demonstrate: 1) localized paravertebral longitudinal cartilage/bone accumulations (PVLC/BAs) invariably associated with branched, fused and asymmetrically spaced ribs that emanate from it laterally; 2) abnormal rib formation immediately adjacent to abnormal vertebral body and intervertebral disc formation in asymmetric right/left fashion; and 3) patterns of rib deformation that differ in each mouse. Normal BALB/c embryo and age-matched non-affected *pu/+* mice assessments allow for *pu/pu* comparisons. The *Dll3* Notch family gene is involved in normal somitogenesis via the segmentation clock mechanism. Although pathogenesis of rib deformation is initially triggered by the *Dll3* gene mutation, these findings of abnormal asymmetric costo-vertebral region structure imply that differing patterns cannot be attributed to this single gene mutation alone. All findings implicate a dual mechanism of malformation: the *Dll3* gene mutation leading to subtle timing differences in traveling oscillation waves of the segmentation clock and further subsequent misdirection of tissue formation by altered chemical reaction-diffusion and epigenetic landscape responses*.* PVLC/BAs appear as primary supramolecular structures underlying severe rib malformation associated both with time-sensitive segmentation clock mutations and subsequent reactions.

## Teaser

**Summary:** Whole-mount preparations and serial histologic sections in pudgy (*pu/pu*) mice demonstrate localized paravertebral longitudinal cartilage/bone accumulations (PVLC/BAs) invariably associated with branched, fused and asymmetrically spaced ribs emanating laterally from them.

## INTRODUCTION

Rib deformities are intimately associated with vertebral body and intervertebral disc abnormalities in many developmental mouse and human disorders. The vertebral and disc abnormalities lead to spinal deformity and shortening (congenital scoliosis) but the associated rib deformations are in effect the primary problem, restricting chest wall growth and expansion, diminishing pulmonary function and further worsening the scoliosis by asymmetric tethering of spinal growth (thoracic insufficiency syndrome). Each of these malformations occurs in the pudgy mouse, identified as a recessive gene disorder (Grüneberg, 1961); and subsequently attributed to a mutation of the *Dll3* gene of the Notch group (Kusumi et al., 1998). Several Notch pathway genes underlying the segmentation clock controlling normal embryonic somitogenesis have been identified (Dequéant and Pourquié, 2008; Krol et al., 2011; Saga, 2012). Mutations in the *Dll3* and other segmentation clock pathway genes have been demonstrated to lead to axial malformations in both mouse and human disorders of the spondylocostal dysostosis skeletal dysplasia group (Turnpenny et al., 2017; Nóbrega et al., 2021; Umair et al., 2022). While both the normal molecular pathways of the segmentation clock and mutations in the *Dll3* and other Notch pathway genes leading to axial malformations have been outlined in detail in numerous studies, specific supramolecular structural studies of the step-by-step pathogenesis of the malformations have not been done. The term ‘supramolecular structure’ refers specifically to tissue or anatomic conformations where the primary gene/molecular components of the normal structure and/or the gene mutations causing abnormal or malformed structure are known. The subsequently formed structure is then considered as being associated with or beyond the specific normal/abnormal molecules in the developmental hierarchy and is thus defined as the supramolecular structure. Our previous structural study in the pudgy mouse concentrated on the abnormal vertebrae and discs characterized by fused vertebral bodies (block vertebrae or unilateral bars), wedge vertebrae, hemi-vertebrae and bifid vertebrae as well as absent, partial or markedly misshapen and mal-oriented intervertebral discs (Shapiro, 2016); this study concentrates on the rib malformations.

We have identified abnormal paravertebral tissue collections in the pudgy (*pu/pu*) mouse and refer to them as paravertebral longitudinal cartilage/bone accumulations (PVLC/BAs). These focal abnormal embryonic accumulations, caused by a mutation in the Notch pathway *Dll3* gene, appear as the primary component of abnormal rib development from which the branched, fused and asymmetric rib malformations emanate. The paravertebral longitudinal cartilage/bone (C/B) accumulations are initially mesenchymal cell condensations that subsequently become cartilage and then bone via endochondral ossification. While our description is based on these focal accumulations in the pudgy (*pu/pu*) mouse, PVLC/BAs appear to be the primary and essential pathogenic developmental focus from which abnormal ribs emanate in relation to an altered segmentation clock. The tissue paravertebral accumulations associated with developmental rib abnormalities are seen: in several mouse mutations in the Notch pathway segmentation clock oscillation cycle leading to vertebral and rib malformations; in analogous mutations across the spectrum of human spondylocostal and spondylothoracic dysostoses; and in cases of mouse and human congenital axial abnormalities documented in descriptions prior to specific molecular diagnoses but clearly within the spectrum of spondylocostal skeletal dysplasia syndromes.

## RESULTS

### Normal mouse rib, vertebral body and intervertebral disc development: BALB/c mice (10.5 to 16 day pc embryos) and non-affected ***pu/+*** mice (17/18 day pc embryos and newborn to 7-day-old postnatal)

#### Whole-mount observations

**Fig. 1.**
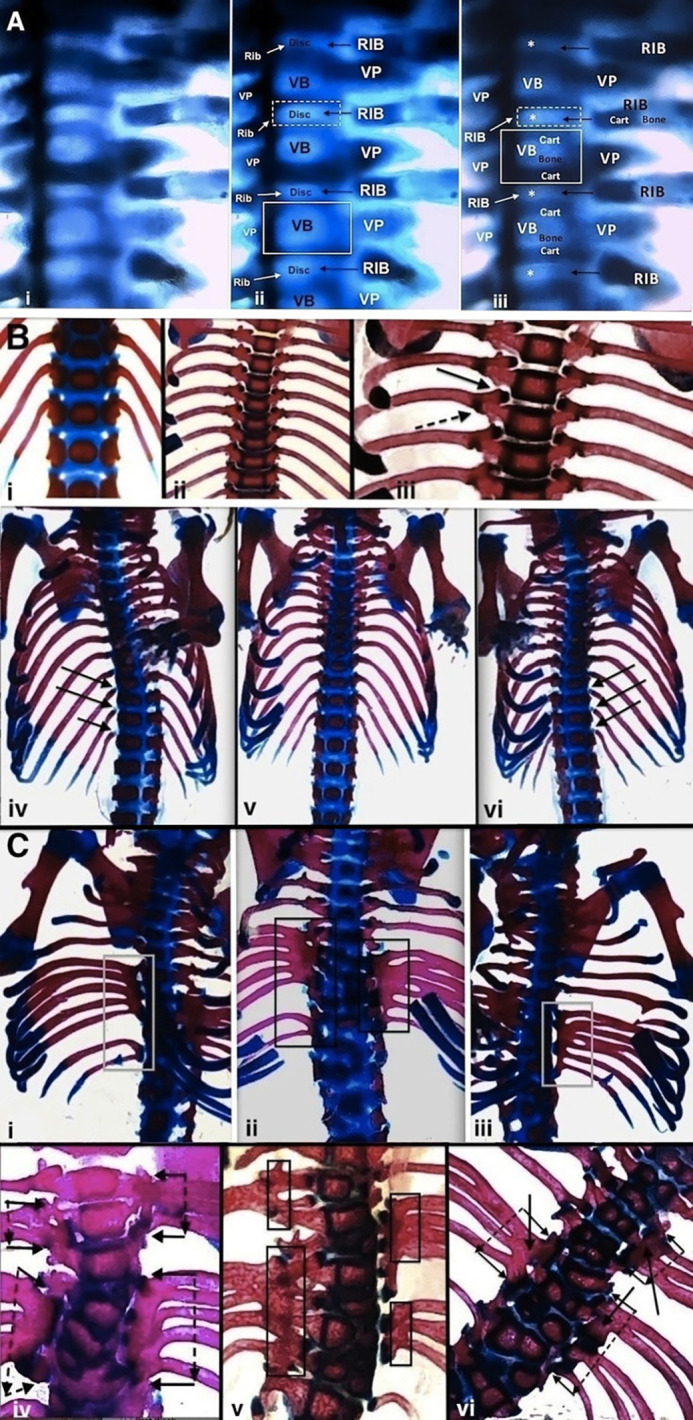
**Whole-mount preparations of vertebral columns and ribs are shown.** (A–C) Whole-mount preparations of vertebral columns and ribs are illustrated. (Ai–iii) Whole-mount images of normal embryonic BALB/c mouse at 16 days pc. Alcian Blue stain shows cartilage tissue as deeper blue with bone and intervertebral disc tissue lighter blue. Images i, ii, and iii are repeated with labeling highlighting different developmental features. The cartilage of the proximal rib aligns linearly with the developing intervertebral disc. VB, vertebral body; VP, vertebral process; both * (white) and Disc, intervertebral disc; Cart, cartilage. (Bi–vi) Whole-mount preparations show normal non-affected (pu/+) spine and rib development. (i) Newborn; (ii) 3-week-old postnatal; (iii) 3-week-old postnatal. Costo-vertebral relationships are clearly delineated. Solid arrow, costo-central joint articular surface of proximal rib (dark blue) relating to facet at postero-lateral region of superior vertebral body and centered adjacent to intervertebral disc; interrupted arrow, costo-tubercular joint. Inferior ends of scapulae are seen at top. (iv–vi) Newborn mouse (three views); (iv) rotation to right side highlights proximal rib cartilage in blue (three arrows) at costo-vertebral joints; (v) antero-posterior projection; and (vi) rotation to left side highlights proximal rib cartilage in blue (three arrows) at costo-vertebral joints. [Fig. 1Bi reprinted from Shapiro, F. (2016) *Adv Anat Emb Cell Biol.* **221**, 1-123, with permission RightsLink/Springer Nature.] (Ci–vi) Whole-mount preparations show abnormal pudgy (*pu*/*pu*) mouse spine and rib development. (i–iii) Newborn pudgy mouse (three views); (i) rotation to right, (ii) antero-posterior view, (iii) rotation to left. Paravertebral longitudinal cartilage/bone accumulations (PVLC/BAs) are indicated by rectangles. Multiple ribs emanate from outer lateral region of each accumulation. Oblique views (gray rectangles i, iii) show clear spacing between PVLC/BAs and vertebral column. Antero-posterior view (black rectangles in ii) appears to show PV accumulation continuity with vertebrae, but this finding is due to overlap since the costo-vertebral joints are at the postero-lateral regions of the vertebrae. (iv–vi) Pudgy mice are each 4 weeks old postnatal. PVLC/BAs are outlined by arrows in iv and vi and by solid rectangles in v. Vertebral body malformations are extensive in each image. Multiple ribs emanate from outer lateral region of each accumulation. Figure v is slightly oblique showing, right side, spaces between the central vertebrae and the irregular articular components (dark blue) of the two PVC/BAs (outlined by solid rectangular boxes). (vi) Solid single arrows entering each rectangle point into paravertebral accumulations.

Whole-mount preparations in seven normal embryonic BALB/c mice at 16 days pc show the cartilage model of the proximal rib to be linearly aligned with the intervertebral disc with a close relationship to the immediately adjacent cartilage models of the developing vertebral bodies (Fig. 1Ai–iii). With further development, the cartilage model of each rib, while remaining opposite the intervertebral disc region, relates more directly to the postero-lateral edge of the vertebral body above (superior) and below (inferior) and to the adjacent vertebral process in newborn to 4-week-old postnatal non-affected *pu/+* mice (Fig. 1Bi–vi) as specific costo-vertebral joint formation continues. Significant deviations from normal are seen in whole-mount preparations from pudgy mice (Fig. 1Ci–vi).

#### Histologic observations. BALB/c mice

**Fig. 2.**
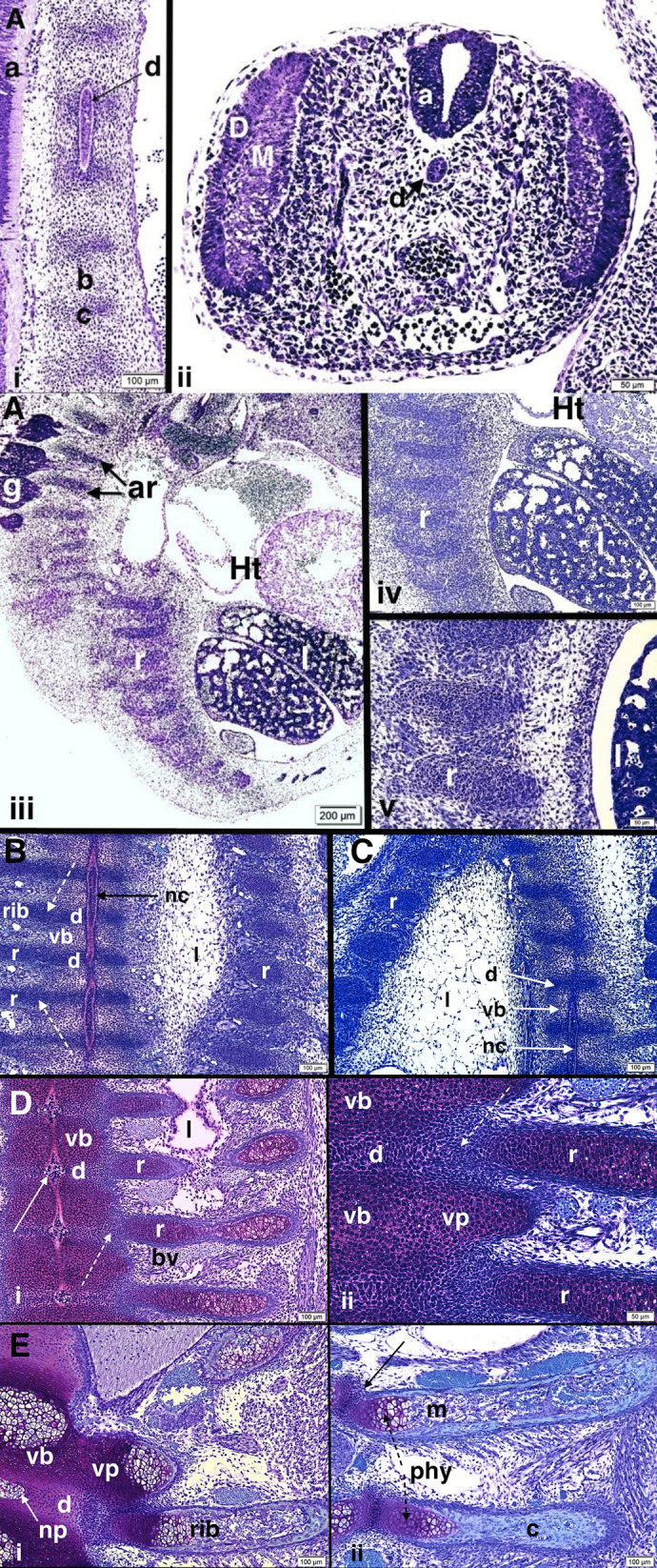
**Normal histologic development of axial skeleton in BALB/c mouse embryos from 10.5 days to 15.5 days pc is illustrated.** (A–E) Normal histologic development of axial skeleton in BALB/c mouse embryos from 10.5 days to 15.5 days pc is illustrated. (Ai,ii) 10.5-day pc BALB/c embryo. (i) Midline section cut in sagittal plane shows portion of notochord (d) along the long vertebral axis surrounded by light-staining cellular condensation of pre-chondrocytes (b) that will form the vertebral bodies and darker-staining cells (c) at intervertebral disc regions. At far left, a portion of neural canal tissue (a) is seen. Scale bar: 100 μm. (ii) Cross-section of embryo, distal to the tissue section in i, shows the neural canal (a) dorsally, notochord (d) below, dermomyotome (DM) at right and left sides and undifferentiated sclerotomal cells surrounding the notochord. Aorta is seen below the notochord filled with blood cells. Scale bar: 50 μm. (iii–v) 10.5-day pc BALB/c embryo. (iii) Parasagittal (dorso-ventral) section (slightly displaced from midline) shows ganglia (g) and cellular condensations that will become vertebral arches (ar) at upper left, cellular condensations that will become ribs at lower central region (r) as well as heart (Ht) and lungs (l); (scale bar: 200 μm). Images iv (scale bar: 100μm) and v (scale bar: 50μm) show developing ribs (r) at progressively higher magnifications. Images highlight developing ribs in cellular condensation pre-chondrocytic stage of development. (B) 11.5-day pc BALB/c embryo. Coronal section, slightly oblique, with developing lung (l) at right, notochord (nc) in long axis centrally surrounded by early cartilagenous vertebral bodies (vb) and more densely cellular, darker staining regions that will become intervertebral discs (d). At left, dense cellular condensations of developing ribs are continuous with cells of intervertebral disc regions (interrupted white arrows). Rib tissue cell condensations are also seen at far right (r). Scale bar: 100 μm. (C) 12.5-day pc BALB/c embryo. Parasagittal section, cut slightly obliquely, with developing lung (l) at left; at right, notochord (nc) along the long axis surrounded by early cartilagenous vertebral bodies (vb) and more densely cellular and darker staining regions that will become intervertebral discs (d). At far right, portions of ganglia are seen. Scale bar: 100 μm. (Di,ii). 14.5-day pc BALB/c embryos. (i) Vertebral bodies (vb) are cartilagenous. Notochord is resolving but at intervertebral discs (d) above and below it develops a diamond shape (solid white arrow) as it transitions to the nucleus pulposus. Ribs (r) continue linear alignment with intervertebral discs and relate to adjacent vertebral bodies above and below with cellular continuity (cellular interzone) between all structures (interrupted white arrow). At central/lateral portions of rib, there is endochondral ossification (chondrocyte hypertrophy) and cellular periosteum forming rib cortex is evident. Bv, blood vessel; l, developing lung. Scale bar: 100 μm. (ii) Magnified view of costo-vertebral development with cellular interzone (interrupted arrow) continuity linking vertebral bodies, intervertebral disc and rib. Scale bar: 50 μm. r, rib; l, lung; vb, vertebral body; d, intervertebral disc; vp, vertebral process. (Ei,ii) 15.5-day pc BALB/c embryo. (i) Vertebral body (vb) cartilage is undergoing endochondral ossification (central chondrocyte hypertrophy). Intervertebral disc (d) is forming with the nucleus pulposus (np) a remnant of the notochord. Vertebral processes of the arches (vp) with chondrocyte hypertrophy are seen; one continuous with the vertebral body and another (upper right) separate and adjacent to the spinal cord (sc). Rib cartilage remains continuous through an interzone region with the periphery of the intervertebral disc and the upper and lower margins of the vertebral bodies. Rib bone formation via endochondral growth and ossification is seen adjacent to the body and disc; a central bone marrow cavity has formed along with rims of cortical bone formed by periosteal osteoblasts. Scale bar: 100 μm. (ii) Two developing ribs are seen. Cellular continuity with the adjacent vertebral bodies persists (solid black arrow). The upper rib shows marrow (m) cavitation while the lower rib has been sectioned more peripherally through continuous cortical bone (c). Physes (phy) are forming in both ribs (interrupted black arrow). Scale bar: 100 μm.

Normal developmental findings in 12 BALB/c mice (two in each time group) from 10.5 days to 15.5 days pc are illustrated in Fig. 2A–E. At 10.5 days pc the embryonic axial skeleton has reached the stage of segmented mesenchymal tissue with alternating dense and relatively loose accumulations of undifferentiated cells, which will become the intervertebral discs and vertebral bodies, respectively, surrounding the continuous notochord in cervical and thoracic regions (Fig. 2Ai). On cross-section of the embryo more distal to the thoracic region the neural canal is well developed, the dermomyotome is seen and sclerotomal cells surround the notochord (Fig. 2Aii). Sagittal sections beyond the midline show heart and lung development to be more advanced relative to the skeletal system (Fig. 2Aiii–v). The developing ribs appear as mesenchymal cell condensations without cartilage differentiation at 10.5 days pc and remain, as cellular condensations, continuous with the relatively dense intervertebral disc tissue accumulations at 11.5 days pc (Fig. 2B) and 12.5 days pc (Fig. 2C). By 14.5 days pc the developing vertebral bodies and proximal ribs have undergone cartilage differentiation but cellular continuity between the proximal/posterior ribs and midline structures persists via continuous interzone regions (Fig. 2Di,ii). The notochord within the vertebral bodies is thinning while it expands into the intervertebral disc regions (becoming the nucleus pulposus), there is chondrocyte hypertrophy within the ribs and the rib periosteum is thickening into a periosteal bone precursor tissue. At 15.5 days pc (Fig. 2Ei,ii) the continuous cellular interzone region persists between ribs, discs and vertebral bodies but there is now chondrocyte hypertrophy at the central parts of the vertebral bodies, at the vertebral (arch) processes and also within the ribs where a paravertebral physis is established. The ribs show periosteal bone synthesis and vascular invasion of the interior leading to hypertrophic cell resorption and bone marrow formation.

#### Pu/+ mice

**Fig. 3.**
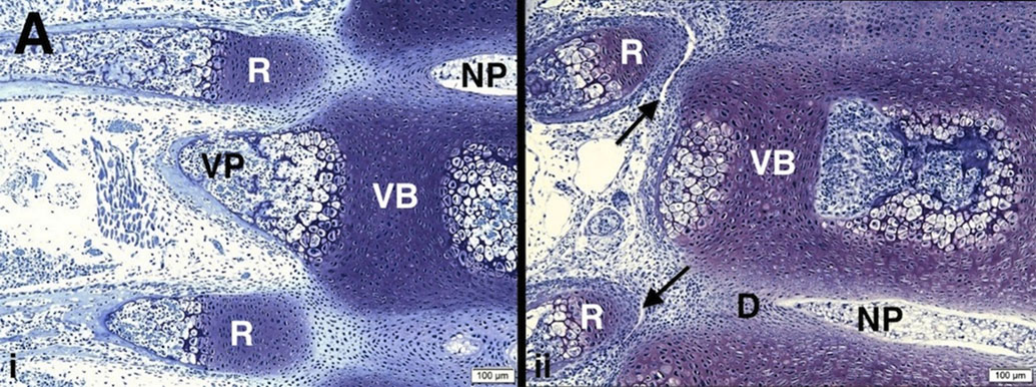
**(A) Vertebral column and rib development in normal non-affected pudgy (*pu*/+) mouse is illustrated.** (i,ii) Normal non-affected *pu*/+ mouse. (i) *pu*/+ newborn. Normal thoracic vertebrae and adjacent ribs are shown. Vertebral body (VB) cartilage is present with vertebral body bone forming at right via chondrocyte hypertrophy and vertebral process (VP) bone at left also via chondrocyte hypertrophy. The nucleus pulposus (NP) of the intervertebral disc is well formed. Rib formation (R) is occurring but cellular interzone tissue still persists at the costo-vertebral region. Scale bar: 100 μm. (ii) *pu*/+ 6-day postnatal. There is early costo-vertebral joint formation with interzone resorption (cavitation) (arrows) at some levels. The nucleus pulposus (NP) of the intervertebral disc (D) is well formed. Scale bar: 100 μm.

Normal developmental findings in newborn (Fig. 3Ai) and 6-day post-natal (Fig. 3Aii) *pu/+* mice are illustrated with considerable maturational changes in the intervening days. In summary: 1) normal BALB/c embryonic ribs at the mesenchymal condensation stage (11.5 days pc) are continuous with the cells of the intervertebral disc but are distinct from them by 14.5 days pc with presence of the cellular interzone; and 2) normal *BALB/c* embryonic and non-affected pudgy (*pu/+*) proximal ribs at the cartilage stage (from 14.5 days pc onwards) develop separately from the intervertebral discs and cartilage models of vertebral bodies and processes but relate closely to them via continuous cellular interzones into the early post-natal period (Figs 2Di,ii,Ei,ii, 3Ai). Beginning costo-vertebral joint formation is seen at 6 days postnatal in occasional sections (Fig. 3Aii).

### Abnormal pudgy mouse (*pu/pu*) rib, vertebral body and intervertebral disc development: 17/18-day pc embryos and newborn to 7-day postnatal mice

#### Comparative findings of rib, vertebral body and intervertebral disc abnormalities in entire pudgy mouse group

In the 37 pudgy (*pu/pu*) mice studied rib cage abnormalities and vertebral and intervertebral disc abnormalities are present in each mouse. However, the structural rib changes (as well as the structural vertebral and intervertebral disc changes) show a different pattern in each mouse. The changes in each mouse are also invariably asymmetric (right versus left sides) with mirror-image changes of paravertebral accumulations and abnormal rib patterns not seen. These variable changes are present, therefore, both in pudgy (*pu/pu*) mice at different ages (from different litters with different mothers) and in pudgy (*pu/pu*) mice born at several specific ages (from the same litter with the same mother). In the 37 mice, these assessments were made in seven who had whole-mount preparations, 30 who had multiple or serial light microscopy sections, and 19 who had full body specimen radiographs. [Most mice had multiple studies done; radiographic and vertebral/disc findings were reported in detail previously (Shapiro, 2016).]

#### Whole-mount observations

Abnormalities of the ribs in affected mice include decreased numbers of ribs (deleted ribs), fused ribs and branching ribs separated by abnormal asymmetric spacing (widening or narrowing). The abnormal fused, branching and asymmetrically spaced ribs invariably emanate from the outer lateral regions of PVLC/BAs. Characteristic continuous paravertebral longitudinal cartilage/bone accumulations generally span two – five vertebral body lengths; from these collections two to three fused ribs and six branching ribs form. In an affected mouse, right and left side abnormalities are invariably asymmetric; they do not occur at the same levels or have the same sizes and shapes. The accumulations are randomly positioned on either side of the vertebral column and intervening areas often display normal (or close to normal) ribs with normal (or close to normal) vertebral body, intervertebral disc and costo-vertebral anatomy. The abnormal ribs emanate (branch off) from these accumulations and do not relate directly to facets on the vertebral bodies or processes as normally seen. Fig. 1Ci–vi shows several examples of PVLC/BAs. The congenital rib deformities are almost always immediately adjacent to (at the same level as) abnormal vertebrae (wedge-shaped, hemi, bifid, unilaterally fused or block fused vertebrae) and abnormal intervertebral discs (partially or completely absent, misshapen or obliquely/longitudinally oriented). Conversely, normal or relatively normal ribs are usually present at the same level as normal vertebral bodies and discs. The patterns of rib malformation are invariably different in all affected pudgy (*pu/pu*) mice, including in siblings born from the same pregnancy. The changes are also invariably asymmetric with right side abnormalities differing from those on the left side. Different patterns of vertebral body and intervertebral disc structure are also seen in each affected mouse.

#### Histologic observations

**Fig. 4.**
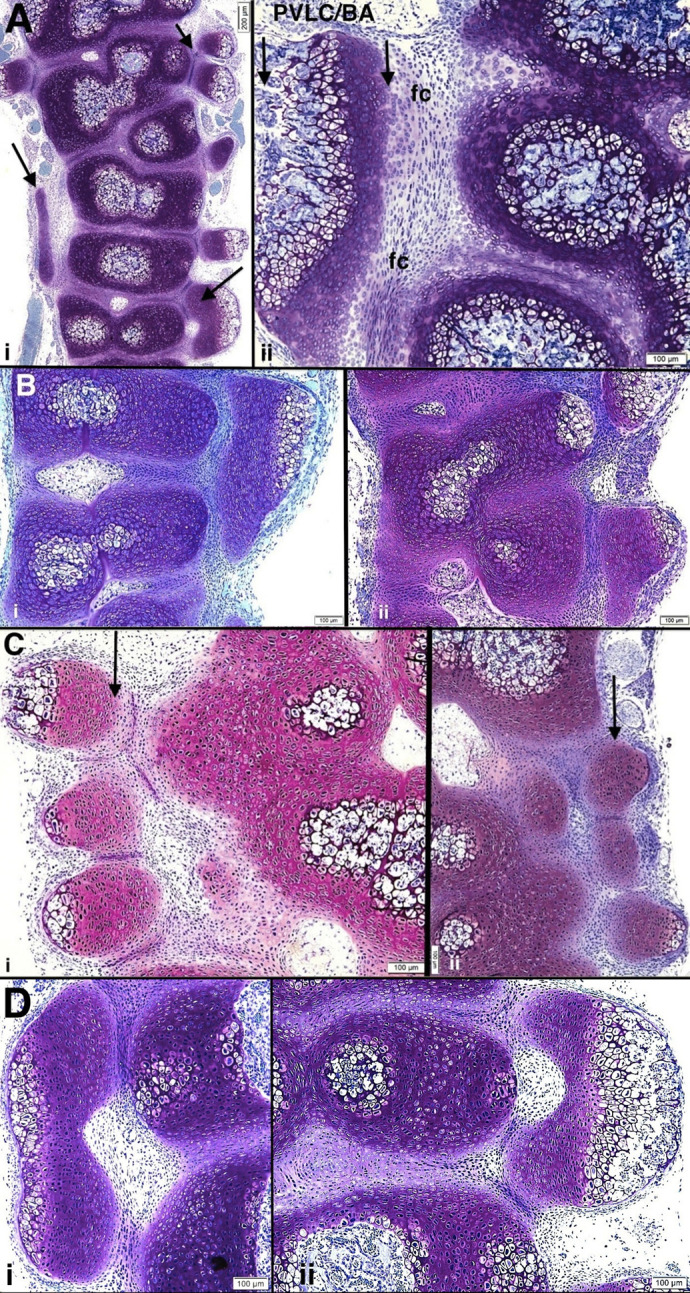
**(A–D) Multiple examples of PVLC/BAs and adjacent abnormal vertebrae, intervertebral discs and ribs in pudgy mice are illustrated.** (i) *pu*/*pu* newborn. Two closely apposed abnormal ribs emanating from a paravertebral cartilage accumulation are seen at upper right (arrow). At lower right and left, additional paravertebral accumulations (arrows) are present. At middle regions ribs are asymmetric, absent or of variable thickness. Scale bar: 200 μm. (ii) *pu*/*pu* 3 days post natal. Abnormal vertebral bodies and intervertebral discs are seen at right with a paravertebral longitudinal cartilage/bone accumulation (PVLC/BA, arrows) at left. The paravertebral accumulation is separated from the vertebrae/discs by a fibrocartilagenous (fc), or sometimes fibrous, tissue. Scale bar: 100 μm. (Bi,ii) *pu*/*pu* 17–18 day pc *pu*/*pu* embryo. (i) Paravertebral cartilage accumulation is seen at right adjacent to and spanning two abnormally shaped vertebrae. (ii) Two paravertebral cartilage tissue accumulations are seen. Scale bars: 100 μm. (Ci,ii) *pu*/*pu* newborn. (i) Three closely adjacent ribs at upper left show (arrow) abnormal shapes and positions. Scale bar: 100 μm. (ii) At right, abnormal paravertebral cartilage accumulation is seen (arrow) from which abnormal ribs emanate further laterally (at deeper sections). Scale bars: 100 μm. (Di,ii) *pu*/*pu* newborn mice. (i) Paravertebral cartilage accumulation is shown at left. It spans two abnormal vertebral bodies and is separated from them by fibrous or fibrocartilagenous tissue. It does not relate to the abnormal disc at all. (ii) A paravertebral cartilage accumulation is seen at right, spanning, in this view, three abnormal vertebral bodies and adjacent to both abnormal intervertebral disc and vertebral body tissue with fibrous tissue interposition between abnormal vertebrae and paravertebral accumulations. Laterally, endochondral ossification is underway. Further laterally, abnormal ribs will emanate from the accumulations. Scale bars: 100 μm.

**Fig. 5.**
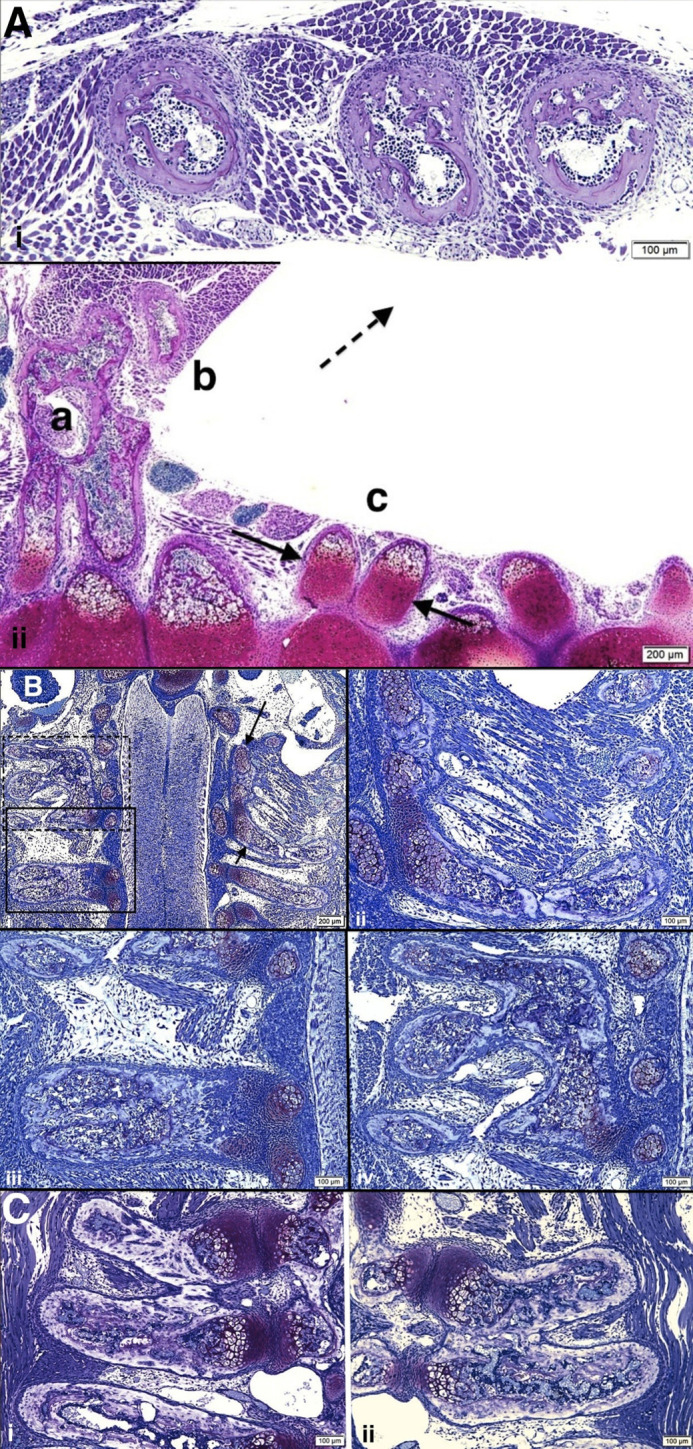
**(A–C) Examples of the variable abnormal rib formation in pudgy (*pu*/*pu*) mice are illustrated.** (Ai) *pu*/*pu* 3 day old postnatal. Peripherally, the paravertebral cartilage/bone accumulation is associated with three markedly malformed and irregularly spaced ribs seen in cross-section. Scale bars: 100 μm. (ii) *pu*/*pu* newborn. Abnormal vertebral bodies, discs and proximal ribs (at bottom) are sectioned along the long axis and the paravertebral cartilage/bone accumulation (PVLC/BA) (a) is cut in an oblique/cross-section orientation. Adjacent to the PVLC/BA (a), a large, abnormally shaped rib is seen (b). Lung tissue was removed (empty space) during tissue processing. Another PVLC/BA is seen (c, between arrows) from which two abnormal ribs emanate. Interrupted arrow points to an area peripherally (not shown) where there are peripheral ribs similar to those as pictured enlarged in i above). Scale bar: 200 μm. (Bi–iv) *pu*/*pu* 17–18** **day pc embryo. (i) Multiple bilateral abnormal ribs emanate from several PVLC/BAs. Developing spinal cord is vertically oriented at center of photomicrograph. Three paravertebral accumulations and their associated abnormal ribs are seen in this image: two at left (solid and interrupted rectangular boxes) and one on right (between arrows). A nerve root extends from a ganglion at left (below lower arrow). The PVLC/BAs and abnormal rib formations from these three regions are highlighted in Bii, iii and iv. Scale bar: 200 μm. (ii) PVLC/BA extends from top to bottom of photomicrograph at right. Cartilage tissue transforms to bone via endochondral ossification. Scale bar: 100 μm. [Fig. 5Bi (reversed) reprinted from Shapiro, F. (2016) *Adv Anat Emb Cell Biol.* **221**, 1-123, with permission RightsLink/Springer Nature.] (iii) Enlargement of solid rectangle in Bi. Two closely adjacent ribs are fused into one large wide rib, emanating from the paravertebral cartilage accumulation. Original cartilage tissue accumulation has been almost completely transformed to bone by endochondral ossification. Scale bar: 100 μm. (iv) Enlargement of interrupted rectangle in Bi. Three ribs emanate from paravertebral tissue mass. Scale bar: 100 μm. (Ci,ii) *pu*/*pu* newborn. Abnormal fused ribs are shown, from the same spine with i left side and ii right side. Again, seen is the separation by flattened fibrous tissue cells of the vertebral cartilage that would normally form the costo-vertebral joint from the paravertebral cartilage accumulation from which the abnormal ribs emanate. Scale bars: 100 μm.

Fused and branching ribs invariably emanate from the continuous PVLC/BAs as noted in the whole-mount preparations above. Fig. 4A–D illustrate histologic structure of the PVLC/BAs and abnormal ribs emanating from them and Fig. 5A–C illustrate abnormal rib structure. The embryonic paravertebral cartilage accumulations and the abnormally positioned ribs progressively transform to bone via the endochondral sequence (explaining C cartilage/B bone reference) and their cortices form via the intramembranous mechanism. Once abnormal rib patterns and the cartilage models of the tissue accumulations are established, the ribs lengthen outwardly (laterally) and ventrally via a physeal mechanism (Fig. 4Ai,ii, Bi,ii, Ci,ii, Di,ii, 5Aii, Bi–iv, Ci,ii).

#### Costo-vertebral relationships

Abnormal ribs do not form initially as continuous tissue extensions from, or with direct anatomic relationships to, cartilage models of adjacent vertebrae; they emanate from the paravertebral tissue accumulations. These paravertebral accumulations invariably show embryonic and early postnatal separation from the adjacent vertebrae appearing as clear spaces on whole-mount preparations. While this finding can be obscured on antero-posterior whole-mount projections due to rib/vertebral body overlap, it becomes evident on oblique whole mount projections (Fig. 1Ci,iii,v). Histologic sections show that the specific costo-vertebral relationships comprising the normal costo-central and costo-tubercular synovial joint articulations do not form. The histologic profile between the PVLC/BAs and the vertebral body/intervertebral disc tissues is generally a fibrous or fibrocartilagenous tissue interposition of variable thickness but some localized areas of imperfect joint formation occur (Figs 4 and 5).

## DISCUSSION

### Normal vertebral and rib development

The structural/histologic outlines of normal vertebral and rib development in the embryonic and early postnatal period have been described over an extended period of time with agreement that the pattern is similar in all amniotes (mammals, birds and reptiles) (Baur, 1969; Verbout, 1985; Scaal, 2016). The vertebrae and ribs form from somites that derive from presomitic (paraxial) mesoderm (PSM). As axial development proceeds studies outline: 1) the axial neural tube and notochord; 2) somitogenesis via the presomitic mesoderm passing from cranial to caudal regions; 3) somite maturation with dermomyotome and sclerotome forming regionally; dorso-laterally the dermomyotome forms skin and muscle tissues and medially/centrally the sclerotome (mesenchymal sclerotome cells) forms the skeletal elements, vertebrae (bodies, arches, transverse processes), intervertebral discs, and ribs*; 4 and 5) more lateral definition of the sclerotome with less cellular cranial components associated with spinal ganglion and nerve formation and denser cellular caudal components comprising arco-costal regions that become the neural arches and ribs respectively; and v) midline differentiation of the perichordal mesoderm forming cell collections destined to become vertebral bodies while the notochord is displaced segmentally to form the nucleus pulposus of the intervertebral discs. [*Sclerotome compartmentalization is further subdivided into: central sclerotome – forming proximal rib (head, neck and tubercle), costo-vertebral joints, neural arch pedicle, transverse process; dorsal sclerotome – forming neural arch lamina, spinous process; ventral sclerotome – forming vertebral body and intervertebral disc; and lateral sclerotome – forming intermediate and distal ribs (rib shafts).]

The orderly embryonic structural formation of ribs, the vertebral column and the costo-vertebral region has been illustrated, with variable degrees of detail, in: garden snake (reptile) (von Ebner, 1888); mouse (Dawes, 1930; von Bochmann, 1937; Dalgleish, 1985; Theiler, 1988); chick (Remak, 1855; Lillie, 1919; Shapiro, 1992; Christ and Ordahl, 1995; Stockdale et al., 2000; Christ et al., 2000, 2004, 2007); gull (Piiper, 1928); sheep (also mouse, human and other vertebrates) (Verbout, 1985); and human (Sensenig, 1949; Töndury, 1953; O'Rahilly and Meyer, 1979; Drews, 1994). Regarding rib development: hox genes control regulation and development of the vertebrae and ribs along the proximo-distal (cranio-caudal) axis with Hox group 6 genes playing a major role in thoracic vertebral and posterior rib development (McIntyre et al., 2007; Vinagre et al., 2010; Scaal, 2021; Khabyuk et al., 2022). All parts of the ribs originate from the thoracic somites initially and subsequently from the sclerotome; further assessments of proximal rib formation then show compartmentalization into the central, medial and ventral/caudal half of the somites/sclerotomes in particular (Verbout, 1985; Huang et al., 1996, 2000; Evans, 2003; Christ et al., 2007; Scaal, 2016, 2021). Some studies suggested that only the proximal ribs originate from sclerotomes with the distal components from the dermomyotomes (Kato and Aoyama, 1998) but other studies, using the same model, confirmed only sclerotomal origin for the entire rib (Huang et al., 2000). Recently, chick embryo rib development study has assigned specific parts of the rib to specific regions and quadrants within the sclerotome (Scaal, 2021; Khabyuk et al., 2022). Vertebrate ribs have three anatomic regions: proximal (vertebral) involving the head (capitulum), neck and tubercle (tuberculum); middle (intermediate) involving the shaft; and distal (sternal) involving the bone/cartilage region that is either independent or directly attached to the anterior sternum (Gray, 1973; Platzer, 2015). These three regions of the ribs arise from three different compartments/quadrants (Aoyama et al., 2005; Scaal, 2021). In the mouse the head of the rib articulates with a facet on the postero-inferior aspect of the adjacent vertebral body immediately above the disc and the tubercle has an articular facet that articulates with the adjacent transverse vertebral process (Bab et al., 2007). [Most human ribs have two articular facets on the head for articulation with postero-lateral vertebral body demi-facets above and below the intervertebral discs (Thane, 1893; Thane, 1894; Gray, 1973).] Costo-vertebral joints encompasses two distinct synovial articulations: 1) costo-central articulation (head of rib engaging with one or two vertebral body facets) and 2) costo-tubercular articulation (between tubercle of rib and transverse process) (Thane, 1894).

In our assessment of BALB/c and non-affected (*pu/+*) mice we detail normal costo-vertebral formation for comparison with abnormal rib development in pudgy (*pu/pu*) mice related to our identification of PVLC/BAs in the *pu/pu* group. Sections in the 11.5 and 12.5 day pc embryos show cellular continuity between the mesenchymal cells of the pre-intervertebral disc, the adjacent vertebral bodies and the proximal rib region along with linear alignment of the developing rib with the developing intervertebral disc and the 16 day pc whole-mount preparation shows linear continuity of proximal rib, developing joint and intervertebral disc, a relationship that persists as development proceeds. These relationships are not widely appreciated and are highly relevant to subsequent abnormal rib development. Throughout normal embryonic and early postnatal development, from mesenchymal condensation to cartilage to bone, there is no structure resembling the abnormal paravertebral cartilage accumulation that we describe in pudgy (*pu/pu*) mice; each rib is structurally independent from ribs forming above and below, is linearly aligned with the intervertebral disc, and relates directly to adjacent cartilage models of the vertebral bodies via cellular inter-zones.

### The PVLC/BA and abnormal rib development as shown in the pudgy mouse

This study in the pudgy (*pu/pu*) mouse identifies three groups of findings, at the cell and tissue level, which have not been clearly defined previously. The supramolecular structural study demonstrates, a) a specific tissue accumulation, referred to as the PVLC/BA, from which the abnormal ribs emanate. These pathologic tissue foci (PVLC/BAs) are invariably directly associated with the abnormal formation of branching, fused and irregularly spaced ribs that accompany the vertebral body and disc abnormalities. The initial cellular accumulation is an embryonic abnormal paravertebral mesenchymal condensation that progressively differentiates to cartilage and then bone via the endochondral sequence, with surface cortical bone formed by the intramembranous mechanism. The abnormal ribs (fused, branching, irregularly spaced and decreased in number) emanate from the outer lateral margins of the accumulation; once established they continue to expand outwardly and ventrally by the endochondral mechanism that forms a physis within the paravertebral cartilage accumulation. The abnormal ribs emanate from the outer lateral borders of the tissue accumulations; they do not relate directly with the vertebral bodies or processes. b) Abnormal paravertebral accumulations and rib formations occurring immediately adjacent to sites of vertebral body and intervertebral disc malformation and in an asymmetric (right versus left) fashion. The PVLC/BAs form immediately adjacent to (at the same level as) vertebral body malformations (wedge-shaped, hemi, bifid or fused) and intervertebral disc malformations (absent, misshapen, oblique or longitudinally oriented). Conversely, normal, or virtually normal, ribs in affected mice are adjacent to normal, or virtually normal, vertebral bodies and discs. In addition, the abnormal PVLC/BAs and the altered ribs are invariably asymmetric, with differing patterns on right versus left sides. These associations imply a direct relationship involving altered segmental clock functions spanning vertebral body/intervertebral disc/proximal rib regions involved and are determined unilaterally. c) Each affected pudgy (*pu/pu*) mouse has a different pattern of paravertebral cartilage accumulations and of rib (as well as vertebral body and intervertebral disc) abnormalities – including in those siblings from the same litter/mother. This indicates that the single gene mutation alone cannot fully account for the malformation findings. The specific developmental cellular details of these three supramolecular structural findings need to be taken into account when establishing mechanisms of ultimate deformity.

### Paravertebral accumulations and abnormal rib formation originate at the somite stage in pudgy (*pu/pu*) mice and in several Notch pathway gene mutation models

The supramolecular structural changes leading to the paravertebral tissue accumulations and subsequent rib malformation are initially evident as somite abnormalities that appear histologically as defective somite patterning with partial to full fusion of adjacent somites. Grüneberg (1961), in his initial description of the pudgy variant, noted abnormal embryonic somite formation beginning at 8 days pc and continuing to 12.5 days pc (“abortive segmentation into somites”). The changes involved somite tissue as irregular and continuous, with a “complete absence of intersegmental fissures” and continuing “non-separation of the sclerotomes”. In pudgy mice with defined *Dll3* mutations (that led to axial vertebral body, disc and rib deformities) histologic abnormalities in one study showed presomitic mesoderm thickened and disorganized; somites irregular in size and shape; and somite borders irregular (not sharply defined) (Kusumi et al., 1998) and in another study there was: paraxial mesoderm with irregularly sized epithelial somites with lack of normal structure; and delayed and irregular somite formation with reduced mesenchymal condensation (cell density) (Dunwoodie et al., 2002). One way that the *Dll3* gene mutation manifests itself is in the disorganized cellular relationships in the somites and peri-somitic cells. As we demonstrate in the pudgy (*pu/pu*) mice in this study, these changes ultimately lead to the PVLC/BAs from which the abnormal ribs emanate. Studies of mutations in other genes of the Notch pathway in mice also show specific histologic examples of adjacent somite fusions (where PVLC/BAs and abnormal rib formation are also seen to occur); these include: *Mesp2* (Saga et al., 1997); *Lfng* (Zhang and Gridley, 1998; Evrard et al., 1998); and *Hes7* (Bessho et al., 2001). In *Mesp2* mutated mice, histology demonstrates defective somite segmentation at 9 and 11 days pc with “lack of clearly segmented blocks (somites)” described (Saga et al., 1997). There is either a lack of somite formation or defective somite patterning in *TBX6* gene mutations (depending on type and severity) (Chapman and Papaioannou, 1998; White et al., 2003) and induced mutations in *RIPPLY2* mice show segmentation abnormalities with irregular dermomyotomes and ill-defined boundaries (Morimoto et al., 2007). In mice with axial vertebral body and disc malformations (prior to identification of the genes responsible), partial or complete fusions of adjacent somites in histologic sections were shown in crooked tail (*Cd*), rachiterata (*rh/rh*), and (*rv/rv*) mice (Theiler, 1988).

### Theoretical and molecular models of morphogenesis: normal models of axial segmentation and their relationship to vertebral and rib formation

Normal axial vertebral-rib formation and limb formation have been intensively studied. Specific temporal sequences of embryonic development and sequential patterns of structural development in various regions of the body have been identified. Axial development passes in a cranial-caudal direction with a clear-cut repetitive segmentation pattern of vertebral body–intervertebral disc–vertebral body and rib–space–rib formation; limb development is characterized by upper extremity development slightly preceding that in lower extremities and a proximal–distal pattern (shoulder/hip regions first proceeding to elbow/knee and then to wrist/ankle and hand/foot). Various theoretical models have been developed to account for these developmental patterns.

#### Theoretical models of morphogenesis.

Turing proposed that a series of chemical diffusion-reaction events help determine morphogenesis (Turing, 1952). Since then, studies have concentrated on further specifying the theoretical and structural components of pattern formation in general and axial segmentation and limb formation in particular. There has been renewed recent interest in developing the principles of the Turing approach, including deepening it from a one- and two-dimensional to a three-dimensional approach (Ben Tahar et al., 2023 preprint). Regarding axial development, two basic models of somite formation have been proposed that ultimately underlie vertebral and rib segmentation. Within this framework of theoretical models, explaining and directing investigation into axial formation, two have predominated: 1) Positional information model, where an anterior–posterior gradient produces confrontation between groups of cells to generate positional information by reaction-diffusion mechanisms leading to a structure formation–boundary–structure formation sequence (Wolpert, 1969, 1989; Gierer and Meinhardt, 1972; Meinhardt, 2008) and 2) clock and wavefront model, where the clock is an intracellular oscillator phase-linking cells throughout the embryo (cellular biorhythms leading to somite formation with a biological clock-like regularity in time) and the wavefront is a kinematic wave of cell change moving slowly down the long axis of the embryo (Cooke and Zeeman, 1976). Interplay occurs between the clock and the wavefront with somitogenic cells expressing properties for differentiation (off and on cycles) only during part of the oscillation cycle leading to alternating patterns. Combination of these ‘two big ideas’ (reaction-diffusion and positional information) is now underway (Green and Sharpe, 2015). Integration of these models with the molecular constituents of the segmental clock in segmentation and regionalization of vertebrate versus insect body plans has been extensively updated recently (Diaz-Cuadros et al., 2021).

#### Molecular models of segmentation

Definitive explanation of structural formation requires knowledge of the associated molecular events. The mechanisms of segmentation involving specification within the paraxial mesoderm to somite formation, subsequent establishment of axial structures and recognition of the various genes involved have been outlined (Keynes and Stern, 1988; Tam and Trainor, 1994; Kmita and Duboule, 2003; Fleming et al., 2015). Normal axial segmentation is now recognized to occur in relation to a segmentation clock; somite pairs are rhythmically produced from PSM at the determination front over 2 h in the mouse (zebrafish 30 min, chick 90 min and human 4–5 h). Additional pairs are added in a cranial-caudal direction. *Dll3* is expressed in the PSM (Kusumi et al., 1998; Bessho et al., 2001; Chapman et al., 2011). It is not present on the cell surface in the PSM but is seen primarily in the Golgi apparatus and adjacent cytoplasmic vesicles (Chapman et al., 2011). There is increasing recognition of the signaling molecules associated with normal segmented pattern formation, many of these being a series of cyclic genes in the PSM with as many as 40–100 identified in mouse, chick and zebrafish with variable distribution in each (Pourquié, 2003; Dequéant and Pourquié, 2008; Krol et al., 2011; Saga, 2012; Onai et al., 2015; Diaz-Cuadros et al., 2021; Weldon and Münsterberg, 2022). Much of the function of the cyclic molecules involves establishment of regularized somite borders (Hrabé de Angelis et al., 1997; del Barco Barrantes et al., 1999). What has come to be known as the segmentation clock involves the Notch family of genes interacting with fibroblast growth factor (FGF) and *Wnt* pathways with additional regulatory control by retinoic acid (RA) (Dequéant and Pourquié, 2008; Krol et al., 2011; Chen et al., 2016). Negative feedback loops are established with cyclic genes of Notch and FGF families oscillating in opposite phases to cyclic genes of the *Wnt* family. The Notch family consists of 30–40 regulatory genes, prominent being delta like-3 (*Dll3*), mesoderm posterior 2 (*Mesp2*), lunatic fringe (*Lfng*) and hairy-and-enhancer-of-split 7 (*Hes7*). Notch pathway cyclic genes include *Hes7* and *Lfng* (lunatic fringe glycosyltransferase), but the Notch pathway gene *Dll3* is not cyclic or expressed periodically in the PSM (Bessho et al., 2001). While most cyclic genes are involved in signal transduction or transcription and belong to the Notch, FGF and Wnt pathways, in the segmentation clock only a subset of the components of these three pathways are expressed in cyclic/periodic fashion (Dequéant et al., 2006; Aulehla and Pourquié, 2008; Dequéant and Pourquié, 2008). Other genes essential for vertebrate somitogenesis include factors involved in regulation of developmental processes in paraxial mesoderm that subsequently interact with Notch family genes: T-box transcription factor 6 (*TBX6*) that encodes a T-box transcription factor that helps activate *Mesp2* and *Dll1* gene expression and *RIPPLY2* that encodes a nuclear transcriptional repressor protein.

### PVLC/BAs are present in many mutant mouse and human vertebral body-disc deformities with markedly abnormal rib deformation

Abnormal ribs have been associated with congenital scoliosis in humans with skeletal dysplasias in skeletal pathology assessments as early as 1836 (Rokitansky, 1836; Nader and Sedivy, 2004); in the initial pudgy mouse description (Grüneberg, 1961); and in studies recognizing that mutations of the *Dll3* gene of the Notch pathway cause the pudgy mouse malformations (Kusumi et al., 1998). While we describe the PVLC/BAs in the pudgy mouse, this abnormal cartilage/bone region is evident (when searched for specifically) in many mutant mouse and human abnormal vertebral-disc/congenital scoliosis disorders associated with branching, fused and abnormally spaced ribs. These observations further demonstrate that the paravertebral accumulation is the primary pathoanatomic structure underlying severe rib malformation associated with temporal segmentation clock mutations. These are revealed by whole mount preparations in mutant mice and combinations of skeletal drawings, plain radiographs and computerized tomography (CT) or magnetic resonance imaging (MRI) in humans. Table 1 presents a summary of PVLC/BAs with rib deformation (accompanying vertebral body and disc abnormalities) that can be identified in whole-mount or radiologic specimens in mouse and human disorders from published material. Table 1 a)i outlines the mouse models and a)ii the human disorders defined by the specific gene mutations of the segmentation clock; b) the PVLC/BAs seen in imaging studies in human cases of well-defined spondylocostal and spondylothoracic dysostoses without molecular studies; c)i the PVLC/BAs in mouse spondylocostal models described in the pre-molecular era; and c)ii the PVLC/BAs in human disorders that initially established the spondylocostal and spondylothoracic skeletal dysplasia entities that have subsequently been shown to be caused by gene mutations in the segmentation clock pathway.

**Table 1.**
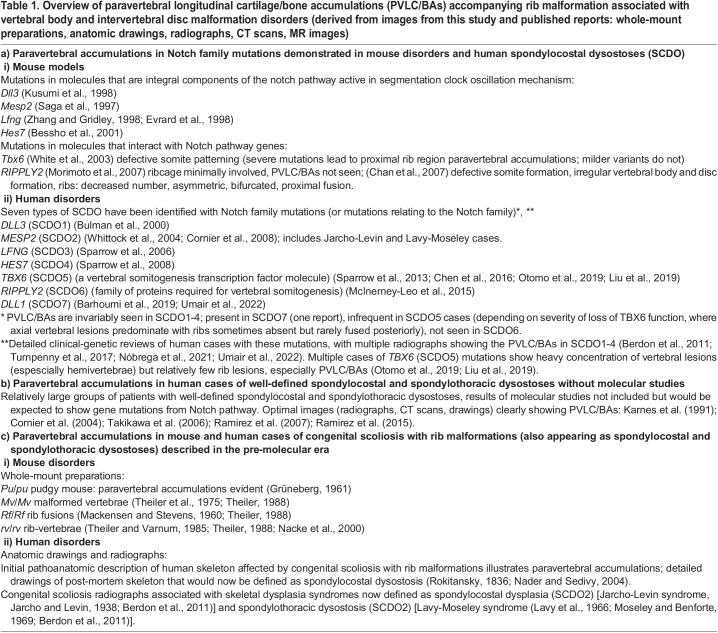
Overview of paravertebral longitudinal cartilage/bone accumulations (PVLC/BAs) accompanying rib malformation associated with vertebral body and intervertebral disc malformation disorders (derived from images from this study and published reports: whole-mount preparations, anatomic drawings, radiographs, CT scans, MR images)

#### Mouse findings

While the PVLC/BAs are clearly defined and prominent in the pudgy mouse due to *Dll3* mutations, they are also very prominent in *Mesp2*, *Lfng*, and *Hes7* mutations, variable in *TBX6* transcription factors that are less directly involved in segmentation (although they help activate *Mesp2* and *Dll3*), and rarely appear or are absent in *RIPPLY2* mutations (that help define segmental borders). It has also been demonstrated that with targeted mutations of the homeobox *Uncx4.1* gene proximal ribs and neural arch pedicles are not formed although PVLC/BAs are not present in the illustrations presented (Mansouri et al., 2000). The *Uncx4.1* gene is not necessary for somite segmentation but serves to maintain condensation of the caudal half of newly formed somites and sclerotomes. As mouse and human cases with spondylocostal and spondylothoracic dysostosis accumulate and undergo detailed gene study, it becomes clear that even within the seven gene-specific mutations affecting the Notch and temporal segmentation pathways currently identified each specific gene is affected by differing types of mutations including nonsense, missense, small deletion and small insertion changes (Cornier et al., 2008; Otomo et al., 2019; Umair et al., 2022). To the Notch pathway (or pathway-related) gene with a mutation causing axial abnormality and the slightly differing temporal abnormalities induced we can also add, as a third cause in many groups, the type and position of the mutation within the pathway gene involved.

#### Human findings

The mutations in the Notch pathway and Notch pathway-related mouse genes that disturb the segmentation clock control of somitogenesis and lead to axial deformation are also found in humans where they lead to skeletal dysplasias associated with vertebral/disc abnormalities (congenital scoliosis) and rib abnormalities (thoracic insufficiency syndrome). In humans, the gene mutations lead to specific groups of disorders referred to variably as spondylocostal dysostosis and spondylothoracic dysostosis, and now primarily as the spondylocostal dysostoses (SCDO); these are: *DLL3* (SCDO1), *MESP2* (SCDO2), *LFNG* (SCDO3), *HES7* (SCDO4), *TBX6* (SCDO5), *RIPPLY2* (SCDO6), and *DLL1* (SCDO7) [Table 1 (aii) with articles included in References]. The pudgy mouse caused by the *Dll3* mutation shows the spondylocostal dysostosis (SCDO1) profile in humans. Human disorders caused by *MESP2* are classified as SCDO2; most are more severe than SCDO1 and lead to a disorder primarily defined as spondylothoracic dysostosis (Cornier et al., 2008). In the more severe spondylothoracic dysostosis, plain radiographs and three-dimensional CT reconstructions show prominent bilateral continuous PVLC/BAs from which all ribs emanate and fan out peripherally, forming a shortened ‘crab-like’ thorax (Cornier et al., 2004; Berdon et al., 2011). Although the two terms, spondylocostal and spondylothoracic dysostosis, have been used interchangeably at times, distinction between the two is clear (Roberts et al., 1988; Karnes et al., 1991; Berdon et al., 2011). In this more severe disorder, the proximal ribs are involved bilaterally from the PVLC/BAs. Histopathologic studies for *MESP2* disorders beyond the fused somite stage are not available but we would expect PVLC/BAs to show extensive bilateral paravertebral cartilage accumulations that subsequently transformed to bone. In whole mount sections of embryonic *Mesp2* mutated mice, extensive bilateral PVLC/BAs are present (Saga et al., 1997). Images from mouse and human reports of vertebral-disc/congenital scoliosis with rib malformations show this tissue accumulation to be an invariable finding from which the abnormal ribs emanate. While histologic structural somite abnormalities are seen throughout the several Notch pathway gene mutations studies, the paravertebral accumulations and widespread rib abnormalities appear invariably in the *Dll3* (pudgy mouse), *Mesp2*, *Lfng* and *Hes7* groups.

### Molecular and supramolecular structural mechanisms underlie abnormal axial segmentation and rib, vertebral body and intervertebral disc malformation

The pudgy mouse is caused by a mutation in the delta-like 3 (*Dll3*) notch molecule that alters the segmentation clock leading to abnormal somitogenesis and sequential changes causing structural malformation of ribs, vertebral bodies, and intervertebral discs (Kusumi et al., 1998, 2004; Dunwoodie et al., 2002; Chapman et al., 2011). The *Dll3* mutation in the human causes spondylocostal dysostosis (SCDO1) also with malformation of ribs, vertebral bodies, and intervertebral discs (Bulman et al., 2000; Turnpenny et al., 2017; Umair et al., 2022). This study defines an abnormal paravertebral tissue accumulation in the pudgy mouse as the central pathoanatomic focus patterning associated rib malformations and also demonstrates that each pudgy mouse has a different pattern of malformation; since each mouse has a different pattern of malformation, the *Dll3* mutation alone cannot account for the entire range of structural findings. The eventual pattern of rib, vertebral and disc abnormalities can therefore be attributed: 1) to a mutation in the specific *Dll3* Notch pathway gene and 2) to slightly differing temporal irregularities induced in the segmentation clock oscillating network that direct the malformation profile into differing patterns based on variable deviations of a few seconds to minutes. Underlying slight timing variations appear to explain the variable morphologic structural patterns (with the same segmentation clock gene mutation) as also projected by alteration of chemical diffusion-reaction sequences (Turing) and the epigenetic landscape (Waddington, 1957; Holliday, 2006; Goldberg et al., 2007; Bhattacharya et al., 2011; Wang et al., 2011; Cortini, et al., 2016). Other than the demonstration by histology of early somite irregularity and fusion, however, step-by-step pathogenesis of supramolecular structural malformation that follows has not been previously outlined. In the process of identifying PVLC/BAs, it becomes evident that these structural abnormalities and associated rib abnormalities begin with abnormal somite formation as early as 8 days pc and are clearly established by the late embryonic period (17–18 days pc). The site of the molecular mutation concentration (primary abnormality) at specific localizations within the somite and sclerotome as well as molecular concentration changes within the abnormal paravertebral tissue accumulations at the mesenchymal cell condensation, pre-cartilage and cartilage phases of pathogenesis should help outline the relationship between specific gene mutations, temporal asymmetry of molecular expression and the earliest phases of supramolecular structural rib malformations. The proximal rib region is primarily affected by the *Dll3* pudgy Notch pathway mutation where tissue accumulation and developmental deformation occur adjacent to the abnormal vertebral bodies and intervertebral discs. Based on studies in normal amniotes, primarily chick and mouse embryos, mutations in genes of the segmentation clock cycle affect posterior rib formation at its site of initial formation in the sclerotome, particularly in the ventral caudal half of the central sclerotome.

### Summation overview and future approaches

Molecular localization assessments for normal vertebral and rib formation at somite and sclerotome stages have been invaluable (Christ et al., 2007; Scaal, 2016; Scaal, 2021; Khabyuk et al., 2022). In addition, overviews at the molecular level tracing how irregularities amongst segmentation clock signaling genes lead to axial abnormalities (referred to as spondylocostal dysostoses or congenital scoliosis) have been outlined (Pourquié and Kusumi, 2001; Schnell et al., 2002; Kusumi and Turnpenny, 2007; Turnpenny et al., 2007; Pourquié, 2011; Makino et al., 2013; Nóbrega et al., 2021). This study in the pudgy (*pu*/*pu*) mouse demonstrates: 1) the specific cell and tissue accumulation from which abnormal ribs emanate, the paravertebral longitudinal cartilage/bone accumulation (PVLC/BA); 2) the paravertebral accumulations and abnormal rib formation immediately adjacent to (at the same levels as) sites of abnormal vertebral body and intervertebral disc formation and in asymmetric (right versus left) fashion implying segmentation clock gene mutation effects acting across vertebral body/intervertebral disc/proximal rib regions and in a unilateral fashion; and 3) each affected pudgy (*pu/pu*) mouse having a different pattern of paravertebral cartilage accumulations and rib abnormalities (as well as differing vertebral body and intervertebral disc abnormalities). This variability indicates that a single gene mutation alone cannot account for all the structural findings. To formally link individual gene mutations and supramolecular structural findings, the effects of perturbations altering stable states and causing temporal alterations affecting the molecular cascade of the segmentation clock in relation to each component will be needed. The structural pathway starts with abnormal somite and sclerotome formation; moves to an assessment of paravertebral tissue accumulations from mesenchymal to pre-chondrocytic to chondrocyte cells; and follows abnormal rib development to late embryonic and early postnatal stages.

## MATERIALS AND METHODS

### Source, distribution, and ages of normal BALB/c mice and pudgy *pu*/+ and *pu*/*pu* mice

Pregnant BALB/c mice from 10.5 to 16 days pc were obtained from Charles River Laboratories, Wilmington, MA, USA. Non-affected *pu*/+ and affected *pu*/*pu* (Pudgy) mice. Pudgy mice obtained for this study were products of pudgy breeding pairs from JacksonLaboratories, Bar Harbor, ME, USA. For the affected pudgy (*pu/pu*) mice, heterozygous unaffected littermates (*pu/+*) served as controls. An affected pudgy mouse (*pu/pu*) can be identified from the late embryonic time period since it is approximately three-quarters the length of its non-affected littermates (*pu/+*) and has a markedly shortened, twisted tail. The post-natal mice were euthanized by intraperitoneal injections of sodium pentobarbital.

Developing axial, rib and vertebral assessments were performed in 19 BALB/c mice (seven for whole-mount preparations and 12 for serial histologic sections) and, 68 mice in the pudgy group: 31 non-affected *pu/+* and 37 affected *pu/pu* age-matched siblings from 17–18 days pc late embryo to 3 months of age postnatal. In the pudgy groups, *pu/+* mice underwent five whole-mount and 26 histology studies and *pu/pu* mice underwent seven whole-mount and 30 histology studies. The large majority of pudgy group mice were assessed from the late embryonic and early post-natal weeks. There were eight sets of births (litters) in which two or more of the sibling littermates were affected, allowing for a comparison of rib and vertebral anomalies in pudgy mice from the same mother and same pregnancy as well as with all other pudgy mice. The investigation was approved by the Institutional Review Board of the Boston Children's Hospital, Boston, MA, USA.

### Whole-mount preparations

Seven embryonic BALB/c mice at 16 days pc were assessed following processing to the Alcian Blue staining stage described below. Examination was performed using a dissecting microscope with images taken from an attached Apple iPhone X (Apple Inc., Cupertino, CA, USA). *Pu/+* and *pu/pu* (pudgy) mice. Twelve mice underwent whole-mount preparation, seven *pu/pu* and five *pu/+.* The method used was adapted from those described previously (Lundvall, 1904, 1905; Simons and Van Horn, 1971; Shapiro, 1992). After removal of skin, heart, lungs and abdominal contents, each mouse specimen was exposed to running cool tap water for 1 h, dried cautiously with paper towels and placed in a fixative-stain solution of absolute alcohol (80 ml), acetic acid (20 ml) and Alcian Blue 8GX (Sigma Chemical Co., St. Louis, MO, USA) (15 mg) for 24 to 48 h. The length of time in solution was determined by the intensity of Alcian Blue staining of the cartilage of the skeleton. The specimen was dehydrated in absolute alcohol for 5 days. Bone tissue staining was performed with Alizarin Red (Sigma Chemical Co., St. Louis, MO, USA; 10 mg Alizarin Red in 1% potassium hydroxide). Staining continued until a red color indicated skeletal parts that had converted to bone. Clearing was performed in Mall solution composed of 79 ml water, 20 ml glycerin and 1 gm of potassium hydroxide. Once clearing of the adjacent soft tissues was completed, the specimen was transferred to glycerin in increasing amounts from 70% to 80% to 90% and then stored in 100% glycerol at room temperature in the dark. Examination assessed vertebrae and ribs using either a dissecting microscope (Carl Zeiss, Germany) equipped with a Contax RT5 electronic SLR system camera (Yashica, Japan) or an Apple iPhone X (Apple Inc., Cupertino, CA, USA).

### Histologic studies

Histologic studies on the axial skeleton were performed on 12 BALB/c mice, 2 each at 10.5, 11.5, 12.5, 13.5, 14.5 and 15.5 days pc. Pudgy mice. In the pudgy group, 56 mice, 30 *pu/pu* and 26 *pu/+* underwent histologic assessment. Following euthanasia and removal of skin, heart and lungs and abdominal contents, the spines and ribcage segments were dissected intact; fixed in 10% neutral buffered formalin for 2 weeks; and transferred to 25% formic acid for decalcification. Once the tissues were soft, they were cut into smaller segments and prepared using the JB4 glycol methacrylate plastic resin kit. The tissues were infiltrated in JB4 medium (Polysciences, Warrington, PA, USA) for several weeks and then embedded in JB4 plastic for sectioning. Sectioning was performed primarily in the coronal (frontal) plane, but additional sections were cut in sagittal and transverse planes. Sections were cut at 5 μm thickness using a Microm HM 350 rotary microtome (Microm International GmbH, Walldorf, Germany) and stained with 1% Toluidine Blue. Serial sections (60–200) were made in two BALB/c mice at each time period and 13 mice from the pudgy group, 11 pudgy (*pu/pu*) mice and two non-affected (*pu/+*) mice. Serial sectioning was done in coronal plane from mid-thoracic to upper sacral vertebrae. Light microscopy was performed on an Olympus BX50 photomicroscope equipped with an Olympus CMOS SC30 digital camera.
